# Contributions of age, gender, body mass index, and normalized arch height to hallux valgus: a decision tree approach

**DOI:** 10.1186/s12891-023-06389-8

**Published:** 2023-04-11

**Authors:** Do-Young Jung, Sung-hoon Jung, Gyeong-tae Gwak

**Affiliations:** 1 Department of Physical Therapy, College of Tourism & Health, Joongbu University, Geumsan, Republic of Korea; 2 Department of Physical Therapy, Division of Health Science, Baekseok University, Cheonan, Republic of Korea; 3 Department of Physical Therapy, Graduate School, Yonsei University, Wonju, Republic of Korea

**Keywords:** Hallux valgus, Normalized arch height, Decision tree

## Abstract

**Background:**

Hallux valgus (HV) is a common toe deformity with various contributory factors. The interactions between intrinsic risk factors of HV, such as arch height, sex, age, and body mass index (BMI) should be considered. The present study aimed to establish a predictive model for HV using intrinsic factors, such as sex, age, BMI, and arch height based on decision tree (DT) model.

**Methods:**

This is retrospective study. The study data were based on the fifth Size Korea survey, of the Korea Technology Standard Institute. Among 5,185 patients, 645 were excluded due to unsuitable age or missing data, and 4,540 (males = 2,236 and females = 2,304) were selected for inclusion in the study. Seven variables (i.e., sex, age, BMI, and four normalized arch height variables) were used to develop the prediction model for the presence of HV using a DT model.

**Results:**

The DT model correctly classified 68.79% (95% confidence interval [CI] = 67.25–70.29%) of the training data set (3,633 cases). The predicted presence of HV based on the DT was verified against the testing data set (907 cases) and showed an accuracy of 69.57% (95% CI = 66.46–72.55%).

**Conclusions:**

The DT model predicted the presence of HV on the basis of sex, age, and normalized arch height. According to our model, women aged over 50 years and those with lower normalized arch height were at high risk of HV.

## Background

Hallux valgus (HV) is a common toe deformity characterized by excessive angulation, rotation, and lateral deviation of the great toe at the first metatarsophalangeal (MTP) joint [1]. Symptoms of HV include poor-fitting shoes, plantar foot pain, and pain in the medial first MTP joint [2]. The resultant deformity causes pain and discomfort, which decreases the physical function of the foot and has been identified as a risk factor for falls in older people [3, 4]. Also, Traumatic events if not adequately treated could result in degeneration of the joint [5, 6]. Therefore, HV causes several effects that are not limited to the feet [3, 7, 8]. Understanding the risk factors of HV is important for its prevention and management [9, 10].

Various intrinsic and extrinsic factors contribute to HV, such as age [10, 11], sex [2, 4, 10, 11], body mass index (BMI) [10], foot morphology [12, 13], and shoe characteristics [12, 14]. HV is more prevalent in women and older adults [11], and is associated with increased BMI and pes planus [2, 10, 15, 16]. Zhao et al. suggested that sex, age, and obesity affect the arch structure [17]. Therefore, interactions between intrinsic risk factors of HV, such as arch height, and sex, age, and BMI should be considered.

The decision tree (DT) is one of the most popular classification techniques due to its capability of presenting results in a simple format and modelling nonlinear relationships [18–20]. Previous studies of the contribution of intrinsic factors, such as age, sex, BMI, and pes planus, to HV progression assumed linear relationships between outcome and predictor variables [2, 21, 22]. The present study aimed to establish a predictive model for HV using intrinsic factors, such as sex, age, BMI, and arch height, based on a DT model.

## Methods

### Study participants and data

The data of this study were based on the fifth Size Korea survey, which were publicly accessible data, of the Korea Technology Standard Institute (https://sizekorea.kr/human-info/meas-report?measDegree=5). Size Korea was a part of the fifth National Anthropometric Survey, and the fifth survey was conducted among 14,200 Korean civilians aged 0–90 years between 2003 and 2004. Foot measurements were performed at ages 10–69 years among 5,185 participants. Among the 5,185 participants, 645 were excluded due to ineligible age or missing data, and a total of 4,540 participants (males = 2,236 and females = 2,304) were included for analysis. The Institutional Review Board (IRB) of Joongbu University approved this retrospective study and waived the requirement for written informed consent (No. JIRB-2022051101–01).

### Foot scan measurements

**Fig. 1:**
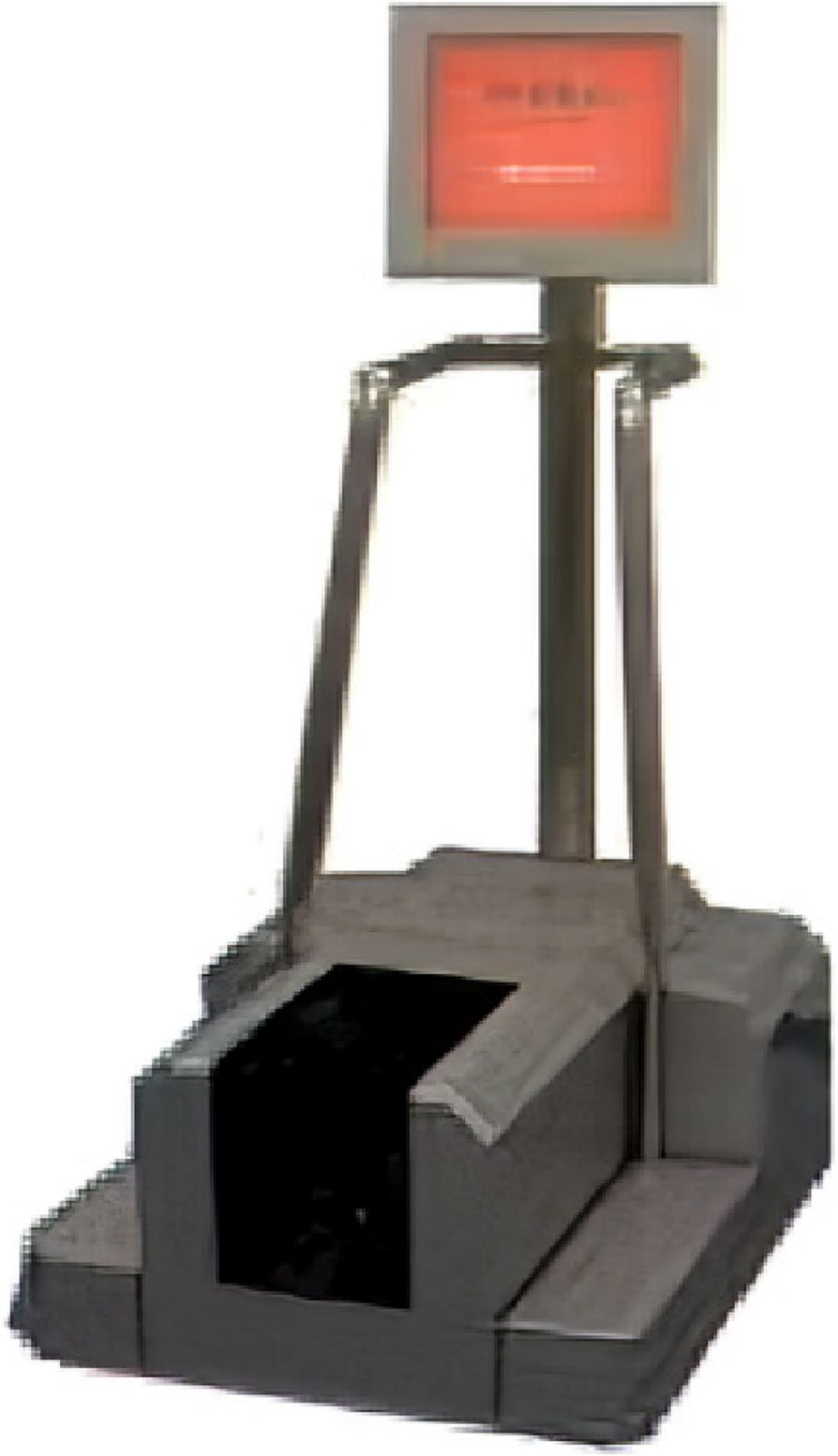
The device used for the measurement of foot scan

During the fifth Size Korea survey, foot scans were performed using the INFOOT 3D Digitizer model IF-21Series (I-Ware Laboratory Co., Ltd, Osaka, Japan) (Fig. 1). ISO 15535 (general requirements for establishing anthropometric databases, 2003) was used to ensure the validity of measurement techniques and data reliability. The 13 non-reflective green velvet markers were placed on specific anatomical landmarks of the foot. Participants were asked to step with the right foot in a resting stance position on to the glass footplate inside the laser scanner, while the left foot was placed next to the scanner on a step of the same height to distribute their body weight equally over both feet. Scans were performed using a scan pitch of 1.0 mm and an optical laser scanning procedure to measure anthropometric data of the foot. Participants were instructed not to move their feet during scanning.

### Data processing

**Fig. 2:**
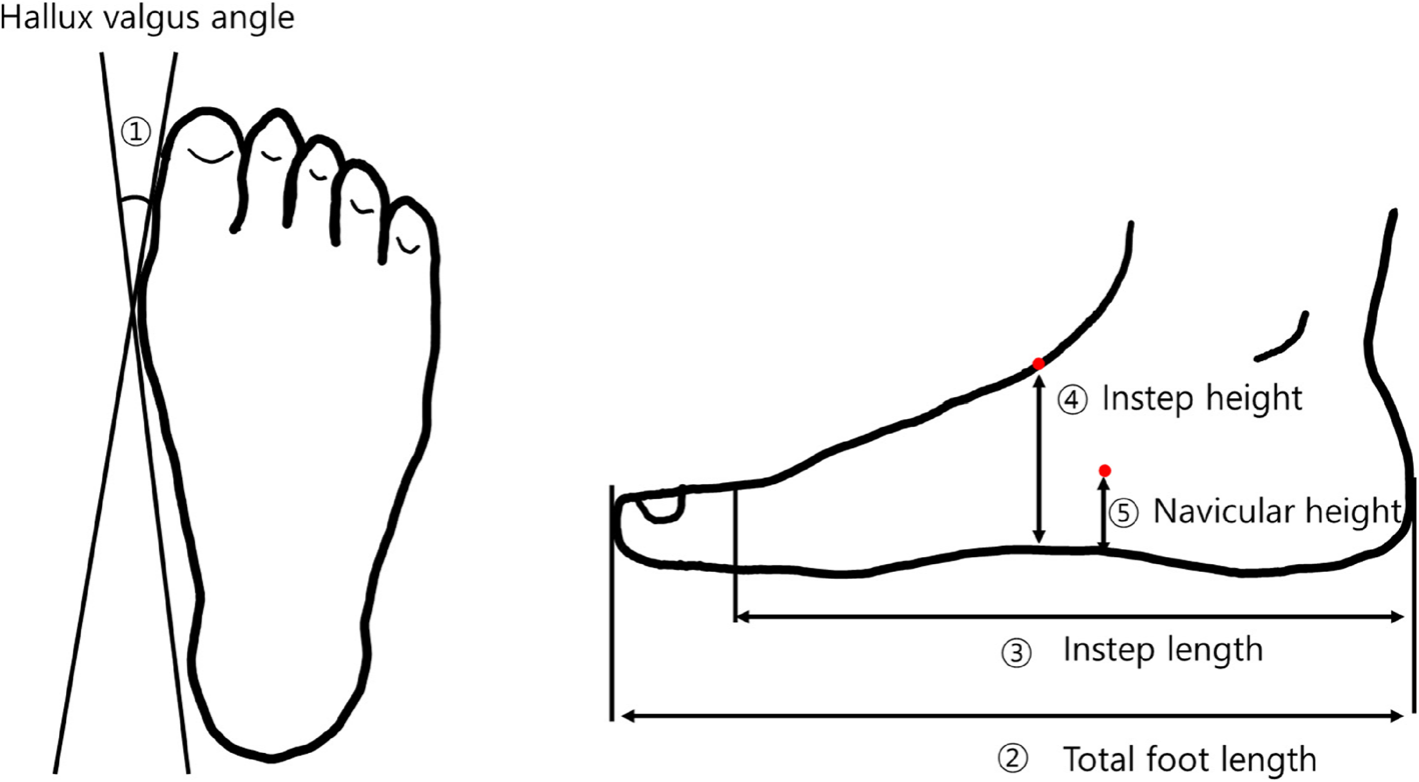
The measurement of foot scan measurement variables

BMI was calculated as body weight divided by height squared (kg/m^2^). The five variables related to foot scan measurements were defined as follows (Fig. 2): First, HV angle (HVA) was calculated as the angle between two lines (one line connecting the medial side of the first MTP joint to the medial side of the heel and the other line connecting the medial side of the first MTP joint to the medial side of the hallux). Participants were assigned to HV (HVA > 10°) and non-HV (HVA ≤ 10°) groups [23, 24]. Second, the total foot length (TFL) was calculated as the distance between the most posterior aspect of the heel and the tip of the longest toe measured along the foot axis. Third, instep length (IL) was calculated as the perpendicular distance between the first MTP joint to the most posterior aspect of the heel. Fourth, instep height (IH) was calculated as the distance between the highest point of the instep and the supporting surface (measurement taken at 50% foot length). Fifth, navicular height (NH) was calculated as the distance between the navicular tuberosity and the supporting surface. We calculated four normalized arch height indices, i.e., NH/TFL, NH/IL, IH/TFL, and IH/IL, from four foot scan measurements.

### Data analyses

The prediction model for HV was constructed using a classification and regression tree (CART) derived using the rpart function of RStudio (version 1.1.463; RStudio, Inc., Boston, MA, USA) based on the R program [25]. Seven variables (sex, age, BMI, NH/IL, IH/IL, NH/TFL, and IH/TFL) were used to develop the prediction model for HV using a CART model. The prediction model used gini impurity to select splits during classification. The subjects from each group were randomly divided into training (80% of subjects) and test (remaining 20%) data sets using ‘createDataPartition’ function of the caret package (http://topepo.github.io/caret/index.html). A total of 3,633 cases were included in the training data set (1,282 cases with HV and 2,351 cases without HV), and 907 cases were included in the test data set (320 cases with HV and 587 cases without HV). The no information rates (classified by chance) were 64.71% in the training data set and 64.72% in the test data set due to imbalanced class.

## Results

**Fig. 3:**
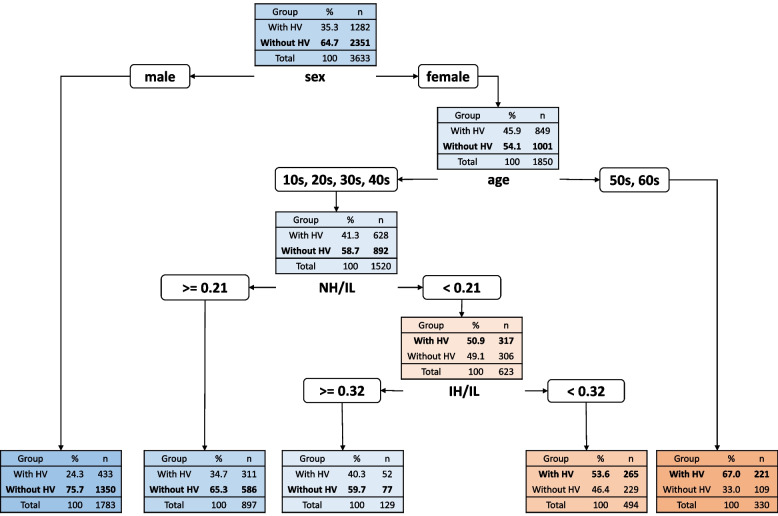
The decision tree model predicting the presence of hallux valgus

Figure 3 shows the DT for HV, which had five terminal nodes. The DT showed that sex was the first predictor of HV, with 75.72% (1350/1783) of the male subjects included in the control group. In the subgroup of subjects who were not males (i.e., female subjects), age was the second predictor. Subjects whose age was not in 10–40 (i.e., aged 50–60) were more likely to have HV (73.67%; 221/330). In the subgroup of subjects who were aged 10–40, NH/IL was the third predictor. The cutoff value of NH/IL for absence of HV was ≥ 0.21. In the subgroup of subjects with NH/IL < 0.21, IH/IL was the fourth predictor. The cutoff value of IH/IL for absence of HV was 0.32. Details of the five divisions with cutoff values of the respective predictors and number of cases classified in each subgroup are presented in Fig. 3.

The DT model correctly classified 68.79% (95% confidence interval [CI] = 67.25–70.29) of the training data set (3,633 cases). The predicted presence of HV based on the DT was verified against the test data set (907 cases) and an accuracy of 69.57% (95% CI = 66.46–72.55%) was obtained (Table 1).

**Table 1:** Performance of the classification tree as calculated during the model training and testing

Calculated evaluation measures		Predicted values		
		Hallux Valgus	Control	
Training data set	Hallux Valgus	796	338	Sensitivity: 37.91%
	Control	486	2013	Specificity: 85.62%
	Overall accuracy (95% CI)			68.79% (67.25, 70.29) %
Test data set	Hallux Valgus	113	207	Sensitivity: 35.31%
	Control	69	518	Specificity: 88.25%
	Overall accuracy (95% CI)			69.57% (66.46, 72.55) %

## Discussion

Previous studies have suggested that three-dimensional (3D) foot scanning could be used for HV diagnosis [23, 26, 27]. Zhou et al. (2013) reported high correlation between HVAs measured on 3D foot scan and radiography (r = 0.70), and that the mean HVA measured by 3D foot scan was 4.9° smaller than that measured by radiography [23]. HV is diagnosed at HVA ≥ 15° on radiography; therefore, in the present study, participants with HVA ≥ 10° on the 3D foot scan were assigned to the HV group.

We included seven variables (sex, age, BMI, NH/IL, IH/IL, NH/TFL, and IH/TFL) in the prediction model for HV. The DT with classification and regression tree selected four variables (sex, age, NH/IL, and IH/IL) from seven variables to predict the presence of HV. Our prediction model for HV selected sex and age as the first and second predictors of HV, respectively, which is consistent with previous studies. Previous studies showed that HV is more prevalent in women and older adults [4, 22]. A meta-analysis of 76 surveys (496,957 participants) reported that the pooled estimate of HV prevalence in females (30%) was 2.3-fold higher than that in males (13%) [11]. In addition, a previous study showed an increase in HV prevalence with age: 7.8% in juveniles (16 studies, *n* = 73,030), 23% in adults aged 18–65 years (15 studies, *n* = 23,790), and 35.7% in older adults (37 studies, *n* = 16,001) [11]. A previous study showed that participants with HV were more likely to be older and female, and have musculoskeletal co-morbidities [28].

Many studies have suggested that pes planus affects the etiology of HV [12, 29]. Despite the commonly held belief that pes planus plays an important role in HV development, the association between navicular height, a measure of pes planus, and HV is controversial [30, 31]. Bryant et al. reported no significant difference in navicular height between the HV group and controls [30]. In contrast, Komeda et al. reported that each point on the medial longitudinal arch in the HV group was significantly lower than the corresponding points in the control group [31]. However, NH and IH cannot be used alone to quantify the pes planus. The roughly triangular shape of the arch indicates a relationship between arch length and height [32]. Therefore, in this study, arch height was normalized by foot length (TFL and IL), to improve the validity and reliability of measurements. In a previous study, NH and IH were divided by TFL and IL, respectively [33]. In the present study, arch heights normalized by TFL (NH/TFL and IH/TFL), and IL (NH/IL and IH/IL) were used to develop the prediction model. However, our prediction model for HV only selected arch heights normalized by IL (NH/IL and IH/IL), which may be because measurement of TFL could be skewed by foot deformities, such as HV and claw toes [33]. Therefore, our results suggest that normalized arch height, especially arch height divided by IL, should be considered when evaluating subjects with HV.

Our prediction model selected NH/IL and IH/IL as the third and fourth predictors of HV with cutoff values of 0.21 and 0.32, respectively. The cutoff values in our model were similar to the results of previous studies [34, 35]. Aboelnasr et al. reported that the optimal cutoff value for diagnosing pes planus was NH/IL ≤ 0.195 [34]. In addition, Hillstrom et al. reported lower IH/IL values in asymptomatic healthy adults with pes planus (0.33 ± 0.03) compared to other foot types, such as pes rectus (0.36 ± 0.03) and pes cavus (0.38 ± 0.03) [35]. Because low normalized arch height is associated with pes planus, our results showed that pes planus would affect the etiology of HV. Considering the node position, our model showed that pes planus could affect the presence of HV in women (aged 10–40 years) but not in men. These results are consistent with the findings of Dufour et al., who reported that pes planus was associated with increased odds of having HV and forefoot pain in women, but pes planus was not associated with HV in men [36]. Similarly, a previous study reported no difference in the odds of having HV based on the presence of pes planus in men, but women with pes planus showed higher odds of having HV than women without pes planus [13]. Therefore, further studies are needed to investigate the risk factors for HV in men.

Previous studies have attempted to predict the risk of lower extremity musculoskeletal disorders [37–39]. Kernozek et al. showed acceptable performances of HV predictive models based on logistic regression of clinical (accuracy of 91.5%) and biomechanical (accuracy of 93.3%) variables [39]. However, the study had a relatively small sample size (40 participants with HV and 51 healthy controls) and did not perform cross-validation. In addition, both models in the study showed high specificity and relatively low sensitivity. Similarly, our model showed high specificity (85.62% and 88.25% in the training and test data sets, respectively) and low sensitivity (37.91% and 35.31% in the training and test data sets, respectively). There are possible reasons for these results. First, the numbers of participants in the HV and non-HV groups were not equal. Therefore, class imbalance could affect the performance of the prediction model. Second, extrinsic and intrinsic factors could contribute to HV development [29]. However, we only used the intrinsic factors, such as sex, age, and pes planus, to predict HV. Therefore, it is necessary to consider the extrinsic factors, such as footwear, occupation, and excess weight bearing, to improve the performance of predictive models for HV. In this study, a predictive model was developed using large number of foot scan data. Our prediction model is easy to interpret, which is one of the strengths of decision tree model [20]. The cut-off value could also be used clinically for evaluation and intervention.

## Conclusions

Our DT model for predicting the presence of HV selected sex, age, and normalized arch height as predictors of HV. According to our model, women aged over 50 years or with low normalized arch height are at high risk for HV. Our model could be used to identify high-risk patients for HV and to recommend conservative management based on the suggested cutoff values.

## Data Availability

The datasets generated and/or analysed during the current study are available in the sizekorea repository, [https://sizekorea.kr/human-info/meas-report?measDegree=5].
